# Granny-Export? The Morality of Sending People to Care Homes Abroad

**DOI:** 10.1007/s11673-021-10118-y

**Published:** 2021-08-10

**Authors:** Bouke de Vries

**Affiliations:** 1 Department of Historical, Philosophical and Religious Studies, Umea University, Umea, Sweden; 2 Centre for Biomedical Ethics and Law, KU Leuven, Leuven, Belgium

**Keywords:** Care homes, Healthcare costs, Migration, Dementia, Lower-income countries, Brain-drain

## Abstract

Many higher-income countries are struggling to make decent and affordable care available to their older populations. In response, some Germans are sending their ageing relatives to relatively high-end care homes within Eastern Europe and South-East Asia where the care tends to be more comprehensive and a lot cheaper. At the same time, this practice has caused much controversy within Germany, with some commentators calling it “inhumane” and “shameful.” The aim of this article is to show that such criticisms are exaggerated. Whereas sending people to care homes in lower-income countries *can be* immoral, I argue that the most promising objections against it do not always apply and, to the extent that they do, do not always provide decisive reasons against sending people abroad. These objections maintain that such expatriations harm three different groups of individuals, namely the emigrants themselves; their friends and relatives; and vulnerable members of the receiving societies.

## Introduction

Many higher-income countries are struggling to provide decent care to their older populations. For example, a study from 2012 found that nurse staffing standards and staffing levels were lower than experts recommended in countries such as the United States, Canada, England, and Germany (Harrington et al., 2012). In a more recent study, health economist Heinz Rothgang found that residents of German nursing homes currently receive an average of ninety-nine minutes of care per day, whereas they are estimated to require an average of 141 minutes in order to enjoy an adequate living standard (Rothgang, 2020). When we take into account that all higher-income countries are ageing and will continue to do so in the coming years and decades (United Nations, 2019)—for example, the share of Germans aged sixty years and above is expected to rise from 27 per cent in 2014 to 35 per cent in 2030 and to 38 per cent in 2050 (Federal Statistical Office of Germany, 2016), whilst the proportion of English people aged sixty-five years and over is projected to increase from 18.2 percent in 2018 to 20.7 percent in 2028 (Office for National Statistics, 2020)—it becomes clear that these problems are unlikely to disappear in the foreseeable future and, indeed, likely to become worse. Not only do the inhabitants of these countries live longer, which comes with a decline in cognitive and physical abilities as well as with a heightened vulnerability to age-related diseases such as dementia, there are increasingly fewer young people to provide them with care as they grow old, with some estimates suggesting that there will be a shortage of nearly half a million care-workers in Germany by 2030 (Rothgang, 2020).

In recent years, some Germans have responded to this situation by sending their relatives to relatively high-end care homes in lower-income countries. Besides being cheaper (Bender and Schweppe 2019; Großmann and Schweppe 2020), these care homes tend to have much better staff-to-resident ratios than in German care homes, which gives their workers more time to look after individual residents (Schwiter, Brütsch, and Pratt 2020; Bender and Schweppe 2019). Although precise figures are lacking, a few thousand Germans are believed to have moved into foreign care homes (Bally-Zenger et al., 2017), which is a number that is bound to increase as the German population continues to age (see the previous paragraph) and as transferring pensions and care allowance from Germany to other countries has become much easier recently, especially to other EU countries (Großmann and Schweppe 2020; Horn et al. 2020). Destination countries are found mainly within Eastern Europe (e.g. Poland, Hungary, Slovakia, Czech Republic) but to a lesser extent also within Southern Europe (e.g. Portugal, Spain) and within South-East Asia (e.g. Thailand) (Bender & Schweppe, 2019; Großmann & Schweppe, 2020; Horn et al., 2020).

At the same time, however, the practice of sending people to care homes within lower-income countries is highly controversial within Germany. On November 12, 2012, the tabloid *Bild* opened with the headline “Increasingly more Germans are being deported [*abgeschoben*] to care homes abroad” (Bild, 2012), whilst Heribert Prantl, a commentator of the *Süddeutsche Zeitung*, wrote during the same year that “Germany should be ashamed of itself” for sending its older inhabitants abroad (Prantl, 2012). Criticisms have also been levelled by Ulrike Mascher, president of the German socio-political advisory group VdK, who said that “we simply cannot let those people who built Germany up to be what it is, who put their backbones into it all their lives, be deported” and by Sabine Jansen of the German Alzheimer Society, who has referred to this type of migration as “inhumane” [*Menschenverachtend*] before adding that Germany should try to “include people with dementia within society, not to exclude them” (Lechner, 2013). A large proportion of the German population appears to share these sentiments. In 2013, a representative survey by TNS Emnida, a German polling institute, found that no less than 85 per cent of Germans are opposed to placing a relative in need of care in a foreign care home “under all circumstances” as opposed to 23 per cent of Germans who are categorically opposed to placing a relative in need of care in a domestic care home (TNS Emnid, 2013).

My aim in this article is to show that such opposition is overdrawn. Whereas sending people to care homes in lower-income countries *can be* immoral, I argue that the most promising objections against it do not always apply and, to the extent that they do, do not always provide decisive reasons against sending people abroad. These objections maintain that such expatriations harm three different groups of individuals, namely the emigrants themselves; their friends and relatives; and vulnerable members of the receiving societies.

Before delving into these matters, a few comments are in order. The first is that, throughout this article, my focus will be on cases where the would-be emigrants have lost the cognitive capacity to decide about moving into a foreign care home, which will usually be due to the fact that they have severe dementia. Under these conditions, and this brings me to the second comment, it becomes necessary for other individuals to decide about their placement within a foreign care home, who may include, for example, a romantic partner, a grown child, a state-appointed guardian, or anyone whom the would-be emigrant has previously nominated as her welfare attorney.

## Harm to Emigrants

The first objection that I want to address here maintains that sending individuals to care homes within lower-income countries is problematic because it would *harm the emigrants*, which is a view that, for example, Sabine Jansen of the German Alzheimer Society endorses when she calls such expatriations “inhumane” (see the previous section). In order to asses this objection, we need to consider what such harm^1^ consists of—insofar as it indeed exists.

### Well-Being

One possibility is that the harm consists of the fact that people receive *worse care* in care homes within lower-income countries than in ones within higher-income countries. However, even if many care homes within lower-income countries are providing worse care to their residents, this does not seem to apply to the care homes to which people from higher-income countries tend to be sent, which are relatively high-end and not rarely recruiting foreign residents specifically. Now, there are no studies that directly compare the care offered by these institutions to the care that is offered by care homes within higher-income countries. Still, there exists evidence, even if a lot of it is testimonial (Schwiter, Brütsch, and Pratt 2020; Bally-Zenger, Eckenwiler, and Wild 2017), that the care that they are providing is often not only not worse than the one available in care homes within higher-income countries but *better* thanks to their superior staff-to-resident ratios. For example, the Polish town of Zabelków has a care home with seventy-five German residents which, according its German manager, can pride itself on a staff-to-resident ratio of one to five or one to six (by comparison, the ratio for people with moderate care needs in Germany is one to thirteen according to estimates by Alzheimer Europe (2020)), as well as on a workforce that is alleged to be as highly trained as staff in German care homes (Großmann and Schweppe 2020; Bender and Schweppe 2019). In a video report by the *Rheinische Post*, several German residents and their family members express high levels of contentment about how the Polish care home is run. For example, August Schmidt, 82, notes how the staff treat them “like their own children,” whereas Horsch Laschet, whose brother is a resident of the care home in Zabelków, says that the “care is done with more love [Liebevoller]” in a way that is “unimaginable within Germany” (Wyglenda 2016; for praise for care homes with German and Swiss clienteles in other lower-income countries, see the quotes in Schwiter, Brütsch, and Pratt 2020, 113–116). One might also consider fieldwork by Jill Brütsch, who in the summer of 2015 spent 2.5 half weeks in Thailand visiting two care homes for German residents with dementia. In both facilities, she found that residents had at least one care worker to themselves at all times (Schwiter, Brütsch, and Pratt 2020, 117).^2^ Even after taking into account that Thai care home staff are not usually as highly trained as their German counterparts (Horn et al. 2020), at least not formally, one would expect that, for some Germans, the downside of receiving care from individuals with lower (formal) qualifications is *outweighed* by the more extensive care than they can receive within these facilities given that, as we have seen in the previous section, the amount of care that is offered by many German care homes is well below the recommended minimum.

Of course, the care that individuals receive within foreign care homes is not the only determinant of their well-being.^3^ Linguistic and cultural differences between sending societies and receiving societies will often matter as well. What this means is that, *even when* better care is available abroad, migrating might still have an overall negative impact upon people’s well-being on account of such differences.

There are a few things to be said in response. The first is that these differences will be small in some cases, if they exist at all. Consider Germans who are sent to areas within Eastern Europe that used to be predominantly populated by ethnic Germans, such as Sudetenland (which is nowadays part of Slovakia and the Czech Republic). Despite the flight and expulsions of many ethnic Germans from these areas after World War II, there continue to be significant German-speaking populations and German cultural influences within some of them (Bally-Zenger, Eckenwiler, and Wild 2017; Großmann and Schweppe 2020).

Second, the sheer fact that there might be meaningful linguistic and/or cultural differences between sending societies and receiving societies does not mean that the language and/or culture of the receiving society, or of a particular substate region thereof, must be unfamiliar to the expatriated. For example, there won’t normally be such unfamiliarity when they have grown up in the area where they are sent.^4^

Third, even when the local language and/or culture is (largely) alien, these differences might not be (fully) manifested within people’s foreign care home. Some Eastern European care homes are trying to attract residents from Germany by hiring bilingual staff, celebrating German holidays, serving German food, offering German television and radio, and having German church services (Bender and Schweppe 2019; Großmann and Schweppe 2020), which are adaptations can go a long way in making these care homes feel like German care homes. Furthermore, insofar as the recruitment of Germans is successful (as it has been in some cases), the residents of these care homes will be able to interact with fellow residents who are likely to have the same mother tongue and share a broadly similar cultural background.

Fourth, any linguistic differences and cultural differences that end up being manifested within foreign care homes might not be (fully) noticed by, or simply be inconsequential for, care home residents with severe cognitive impairments as a result of these impairments (cf. Schwiter, Brütsch, and Pratt 2020; Bender and Schweppe 2019). In particular, it has been suggested that linguistic differences matter little when people with dementia have (largely) lost the ability for verbal communication (cf. Bender and Schweppe 2019) given that, in such cases, non-verbal communication will be a much more important means of communication (e.g. Hubbard et al. 2002; Ellis and Astell 2017).

A fifth and final point that needs to be made here is that any problems of linguistic and/or cultural adjustment that people might face within care homes in lower-income countries will not always have been avoidable within the sending countries. Since many inhabitants of higher-income countries are looked after by foreign live-in caregivers or foreign care home staff, those who remain will frequently encounter cultural and/or linguistic barriers as well (Bally-Zenger, Eckenwiler, and Wild 2017; Borstel 2016).

In short, even when there are linguistic and/or cultural differences between the sending societies and receiving societies, or more specifically between the sub-state regions of the countries between which the expatriated move, this will not invariably undermine the well-being of these individuals. Not only are such differences small in some cases, they are not always (fully) manifested within foreign care homes because of the presence of linguistic and cultural accommodations and, to the extent that they are, not always experienced as alien by the expatriated because they have already lived within the receiving country or not (fully) noticed by them as a result of severe cognitive impairments. Furthermore, any linguistic and cultural obstacles that do exist will not always be (much) greater than the ones that the expatriated would have encountered within domestic care settings given that many care workers within higher-income countries are immigrants.

What about the fact that those who are sent to a foreign care home might end up living further apart from their friends and family members than if they were sent to a domestic care home? The first thing to note here is that not all emigrants will find themselves in this situation. One possible reason for this is that the nearest domestic care home with available rooms is located far away, which may be because there are no domestic care homes in the vicinity of people’s current place of residence (which is especially likely within sparsely-populated areas), as well as because there are simply no affordable domestic care homes nearby, or simply no such care homes with vacant rooms (as might be common in countries with shortages of care home places). Another possible reason is that the foreign care homes to which people are sent are located close to their former place of residence. This may be true, for instance, when they used to live close to Germany’s eastern border, as several Eastern-European care homes with German clienteles are located near this border (Lechner, 2013).

Of course, how easily people can be visited by their friends and family members is not just a function of the geographical distance between them. It also depends upon the available means of transportation and, particularly when it comes to public transportation, the connections between countries are often worse than they are within them. Although this is correct, there are certainly examples of affordable, high-quality public transportation between higher-income countries and lower-income countries; one might think, for instance, of the high-speed train between Vienna and Budapest, which has a journey time of 2.5 hours with tickets starting from 19 euros (see https://www.seat61.com/trains-and-routes/vienna-to-budapest-by-train.htm). And even when public transportation links are poor, car-owning friends and family members might not be (significantly) affected by this, apart from the fact that traveling by plane has become much cheaper in recent decades even if there remain environmental issues with this mode of transportation.

So far, I have suggested that moving to a foreign care home need not always have a (significant) negative impact upon the emigrant’s contact with friends and relatives within the sending society. What I want to add here is that, any reduction in such contact that does take place will normally be *at least partially compensated* by the opportunities for social interaction that exist abroad. For one thing, people who have lived within the receiving society during earlier stages of their life (e.g. during childhood) might have friends and/or family members there to whom they will come to live closer and with whom they might end up having more frequent contact as a result. For another, moving into relatively high-end care homes within lower-income countries may, and often will, provide those coming from higher-income countries with overburdened aged care-systems with more extensive contact with members of staff than they would enjoy within a domestic care home because of the former’s superior staff-to-resident ratios (Horn et al. 2020; Schwiter, Brütsch, and Pratt 2020).

What if the well-being of the expatriated *is in fact undermined* by any linguistic, cultural, and/or social changes that they might experience, and to a greater degree than any linguistic, cultural and/or social changes that they might have undergone had they moved into a domestic care home (insofar as this was an option)? Even in such cases, of which there are clear examples (cf. Horn et al. 2020), it is dubious whether the relevant reductions in well-being will invariably outweigh the increases in well-being that the expatriated might enjoy as a result of living in a specific foreign care home. For given the considerable shortcomings of the aged care within various higher income countries (see the previous section), along with the significantly more extensive care that people can receive within the foreign care homes under consideration, one may reasonably expect that any disutility caused by the unfamiliarity of a local language, culture, and/or social environment will sometimes be *trumped* by the superior care that is available abroad, especially when domestic care homes are struggling to provide basic services to their residents, such as regular showers or baths (cf. Herriger and Schade 2019).

### Autonomy

A critic might concede at this point that when we focus purely on the well-being of the would-be emigrants, there will at least sometimes be decisive reasons for sending severely-cognitively impaired individuals to (relatively high-end) care homes within lower-income countries. However, she may argue that doing so remains morally impermissible on grounds that it is likely to undermine the emigrants’ *autonomy* understood as the extent to which they live more or less successfully in accordance with a conception of the good life (i.e. an understanding of the what makes their life worth living) that they have independently, that is, free from manipulation and brainwashing, endorsed (This conception of personal autonomy draws on Ben Colburn’s conception [2010]). On this view, being sent to a care home within a lower-income country is something that people are unlikely to want upon reflection and, consequently, something that will harm many of them.

One reply here would be that when individuals have lost the cognitive capacity to decide about moving into a foreign care home, they will have lost the ability to live autonomously as they will no longer be able to reflect critically upon what makes their life worth living. To the extent that this is correct, it might be deemed impossible for their autonomy to be undermined by an expatriation.

The problem with this reply is that personal autonomy is commonly, and I assume here correctly, understood to be a property of a person’s *entire life*. What it requires is that someone’s life on the whole converges more or less with her conception of the good life. Whilst such a conception ought to be independently endorsed at *some stage* of her life, which is when she must have the mental competence to reflect upon what makes her life worth living, it is not necessary that she retains this competence *throughout* her life in order to live autonomously. This means, among other things, that even when I am in a permanent coma and therefore no longer capable of reflecting upon what makes my life meaningful or valuable, you visiting me still serves my autonomy when this is something which I would have freely wanted you to do before I entered this perpetual stage of unconsciousness. But if this is correct, then insofar as people did not want, or would not have wanted, to live in a foreign care home when they were still capable of pondering such a scenario, or simply not in one within a (specific) lower-income country, sending them to such a care home will undermine their autonomy.

There are individuals who fit this bill. They will often include those who (used to) have strong patriotic sentiments; those who (used to) have strong attachments to their country’s culture or to one of its cultures; and those who (used to) identify deeply with their country’s natural environment. They might also include those who (used to) have strong wishes to be visited regularly by friends and relatives *regardless* of the cognitive state in which they find themselves, which will in many cases become more difficult once they move into a foreign care home even if this is not always the case (see the previous subsection). What is important for our purposes is that *not everyone* falls into one or more of these categories. In particular, it seems that more cosmopolitan-minded individuals may, and frequently will, not mind being sent to a foreign care home when they can receive superior care there even if this is often on the condition that, every now and then, they continue to be visited by their loved ones. For example, one German resident of the care home in Zabelków reports being “indifferent as to whether one is being looked after by a German or Polish care-giver—the main thing is that one is looked after properly” (Wyglenda, 2016). In fact, sending people to a foreign care home will sometimes be necessary to *honour their autonomy*. This may be the case, for instance, when they have always wanted to spend the final years of their life within their country of origin^5^ or within a country with a warmer and sunnier climate.

What if sending people to foreign care homes does undermine their autonomy? Even then, one might reasonably doubt whether there will always be decisive reasons against expatriating them. At least when sending them abroad is necessary for allowing them to live a minimally decent life—understood as one in which all a person’s basic needs are met, including those to adequate food, shelter, medicine, and care—or simply one that is significantly closer to this threshold, there is a strong case to be made that people’s well-being interests ought to be prioritized over their autonomy interests. To reject this view would suggest that the value of personal autonomy is a master value, i.e. a value that invariably trumps all others. Whilst an evaluation of this claim is beyond this article’s scope, it is worth noting that most philosophers reject it on the ground that it seems to give insufficient weight to other important aspects of human flourishing, including the absence of pain and suffering.

## Harm to Friends and Relatives

Another objection that might be raised against the practice of sending relatives to care homes within lower-income countries is that it harms friends and family members within the sending society—call these “remaining friends and relatives”—by rendering it more difficult and/or costly for them to visit the emigrants.

As with the previous objection, I believe that this objection does not always apply. To vindicate this claim, we should begin by recalling that there are cases where sending people to a foreign care home does not render it (significantly) more difficult or costly for remaining friends and relatives to visit them than if they are sent to a domestic care home, which is especially likely when the nearest available domestic care home is located far away because of a shortage of domestic care home places. Such cases may exist, for instance, when the relevant foreign care home is located just across the border—for example, in 2013, a Czech care home with predominantly German clientele opened at a mere ten kilometres from the German border, close to the Bavarian city of Amberg (Lechner, 2013)—as well as when affordable, high-quality transportation links exist between the would-be sending country and the would-be receiving country—think again of the high-speed train between Austria and Hungary.

But even when expatriating individuals to foreign care homes makes it significantly much more difficult and/or costly for (some of) their friends and relatives to visit them than if they are sent to a domestic care home, this will not always render such expatriations unjustified. One possible reason for this is that costs that the relevant friends and relatives incur under an expatriation are *outweighed* by the costs that they incur if their friend or relative is not expatriated. Whilst this may seem odd, at least when their relationship with the expatriated is not abusive or otherwise dysfunctional (as I assume here it is not), the oddity disappears once we consider that how well people’s lives go is in many cases *intimately linked* to how their friends and relatives are faring due to their love and affection for the latter. Because of these links, it will often be true that when a severely cognitively impaired person can enjoy a significantly better quality of life within a foreign care home, sending her to such a care home will not just promote her own interests but also the interests of any remaining friends and relatives.

There is another way in which such expatriations may, and sometimes will, serve the interests of remaining friends and relatives, namely by relieving these individuals of (perceived) duties to provide *informal care* to their friend or relative. This work, which might involve, for example, helping someone shower, get clothed, get out of bed, and go to the toilet, can, and frequently does, take a substantial physical and psychological toll of people. Just consider a 2016 survey from The Netherlands where one in seven unpaid care-givers, who on average provided twenty-eight hours of care a week, reported that the burdens of their informal care-giving were either “heavy” or “too heavy” (CBS, 2016).

Thus far, my contention in this section has been that sending people with severe cognitive impairments to foreign care homes does not always harm the interests of any remaining friends and relatives that they might have, at least not when taking into account the latter’s full range of interests. What I want to add here is that, *even when* the interests of (some of) these individuals are being set back, this will not invariably count decisively against such expatriations. Especially when moving into a foreign care home is necessary for people with severe cognitive impairments to enjoy a decent standard of living or simply a standard that is significantly closer to this threshold, their interests in being expatriated can be expected to *override* the interests of any friends and relatives in keeping them physically close. To deny this would have implausible implications. For example, it would mean that someone’s interests in living near a friend or parent could trump the friend’s or parent’s interests in having regular showers or baths and in having meaningful daily amounts of social interaction with members of staff. Indeed, it could even trump the friend’s or parent’s interests in not being (regularly) sedated, which has been found to be a pervasive response to challenging behaviour by residents in German care homes (e.g. Gröning and Lietzau 2010; Grassberger and Püschel 2013; Allgemeine Ortskrankenkasse 2017) but is reportedly less common in some foreign care homes with German clienteles (Großmann and Schweppe 2020; Bender and Schweppe 2019).

## Harm to Host Populations

A third and final objection to sending relatives to care homes within lower-income countries maintains that this type of migration harms vulnerable members of the receiving societies. One way in which such harm may arise is that the expatriated take up scarce places in care homes that would otherwise be available to locals. Even if locals do not face formal restrictions upon admission, an influx of comparatively wealthy foreigners might drive up rental prices and render these places out of financial reach of locals who would have otherwise been able to afford them. Another way in which this group may be harmed is that the relevant expatriations engender, or simply contribute to, brain and care drains within the receiving society. This happens when local medical workers and care-workers begin to offer their services to relatively wealthy foreign clients as opposed to members of local population (Bally-Zenger, Eckenwiler, and Wild 2017), which is a phenomenon that has been observed in the related context of medical tourism (Turner, 2007).

What is important for our purposes is that, whilst these are serious problems, they do not seem unavoidable. For starters, insofar as increasingly more people move into care homes within lower-income countries in the coming years and decades—as is likely given that the populations of many higher-income countries are ageing rapidly—states might start taking measures to address any harmful effects of this type of migration for the receiving societies, which would mirror the ones that some states have already taken to address the harmful effects of international brain drains within the medical sector (cf. Snyder et al., 2013). For example, sending states might start building care homes within the receiving countries that partly, if not wholly, accommodate their own citizens so that the number of care home places for locals is not being reduced. Alternatively, or in addition, they might start subsidizing healthcare for the most vulnerable members of the receiving societies. Likewise, receiving states could begin to levy a special tax on care homes with comparatively wealth foreign clienteles and use the revenue generated by this tax to construct affordable care homes across the country and/or to subsidize the medical training of citizens who commit to working in the public healthcare sector for a fixed number of years after their training.

But even when such protective policies are not in place, there remain things that private individuals can do in order to avoid letting this type of migration undermine the health and aged care-access of vulnerable local inhabitants. For example, potential future emigrants might specify in an advance directive that if their relatives ever see it fit to send them to a foreign care home, they should send them to one where their admission is likely to bring benefits to the local population, such as a care home that is struggling to survive due to dwindling resident-numbers. Alternatively, or in addition, they might specify an advance wish that part of the money that they save by living in a foreign care home be donated to local healthcare organizations. And to the extent that people never communicated such wishes before they became severely cognitively impaired, their relatives could still take such protective measures on their behalf if they are legally authorized to do so (I thank Axel Gosseries for alerting me to some of these possibilities).

What if, in spite of any of the macro- or micro-level measures just mentioned, sending people to care homes within lower-income countries remains likely to undermine the aged and healthcare access of local populations? Even then, I do not think that doing so is necessarily morally impermissible. The reason for this is that any harm that such expatriations might cause to the health and general well-being of vulnerable local inhabitants need not be greater than the harm that they would suffer if said expatriations did not take place. To see this, it should be noted that when fewer inhabitants of higher-income countries move to lower-income countries in order to live in foreign care homes, more aged and healthcare workers are likely to move to higher-income countries in pursuit of higher wages. When this happens, vulnerable members of lower-income countries are not rendered better off in any obvious way given that, in this scenario, their aged and healthcare access is undermined as well. In fact, since any friends and relatives of theirs who work as aged and healthcare workers will no longer be living within their country, or only seasonally, it looks like, all other things being equal, it is *morally preferable* for older inhabitants of higher-income countries to migrate to lower-income countries rather than for aged and healthcare workers from lower-income countries to migrate to higher-income countries (cf. Schwiter, Brütsch, and Pratt 2020).

## Concluding Remarks

This article has assessed (what I consider to be) the most promising objections against sending severely-cognitively impaired people to care homes within lower-income countries. According to these objections, such expatriations harm three different groups of individuals, namely the emigrants themselves; their friends and relatives; and vulnerable members of the receiving societies. Whilst each of these objections identifies important potential problems, I argued that these problems do not always exist and that, even when some of them are present, they do not always provide decisive reasons against an expatriation. By way of conclusion, I should stress that I have not tried to offer a list of conditions under which it is morally permissible, if not required, to send people to care homes within lower-income countries, which is something I do elsewhere (see De Vries, Forthcoming). However, if I am right that the force of the objections discussed here is limited, then this provides grounds for optimism that such expatriations can sometimes be morally justified.

## Footnotes

1 I use the term “harm” here to refer to any setback that people might suffer to their interests.

2 Research by Horn et al. (2020) has also found examples of Thai care homes with around-the-clock care, although it is unclear whether they have visited the same care homes as Brütsch.

3 By “well-being,” I mean the presence of pleasure or states of contentment and the absence of suffering and pain.

4 This would be the case, for instance, when Turkish and Moroccan immigrants who have come to Germany and the Netherlands during the 1970s as guest laborers are sent to care homes within their respective countries of origin.

5 It might be asked how it can be known whether severely cognitively impaired people had such wishes. In some cases, they will have explicitly informed others about their desire to be expatriated in old age before they became severely cognitively impaired. Yet even when this did not happen, it may still be possible to infer that this is something that they would want had they still been capable of pondering the matter. This might be the case, for instance, when they have always had strong attachments to their country of origin and are able to able to receive superior residential care within this country. However, since making such inferences can be difficult, I take it that states might need to encourage their citizens to discuss with relatives their preferences about a possible future move to a foreign care home when they are (still) capable of this. I am indebted to an anonymous reviewer for raising this issue.
